# Predicting enantiomer migration order of levobunolol via sequential computational modeling

**DOI:** 10.1007/s00894-026-06809-1

**Published:** 2026-06-11

**Authors:** Pollyanna Pinto Maia, Rafaela Maia Della-Sávia Freitas, Luciana Guimarães, Clebio Soares Nascimento

**Affiliations:** GPQCA: Grupo de Pesquisa em Química Computacional Aplicada, Departamento de Ciências Naturais (DCNAT), Universidade Federal de São João Del-Rei (UFSJ), Campus Dom Bosco, São João Del Rei, 36301-160 Minas Gerais Brazil

**Keywords:** Theoretical calculations, Enantioselective separation, Chiral recognition, Enantiomeric migration order, Levobunolol

## Abstract

**Context:**

A detailed molecular-level rationale for the enantioseparation of the β-blocker levobunolol (BUN) by substituted β-cyclodextrins (β-CDs) is presented through high-level computational modeling. The calculations consistently reveal that the therapeutically active (+)-[S]-BUN enantiomer forms more stable inclusion complexes than the (−)-[R]-enantiomer with both carboxymethyl-β-CD (CM-β-CD) and sulfated-β-CD (SF-β-CD) selectors. Non-covalent interaction (NCI) reveals that the superior stability of the S-enantiomer arises from a more continuous dispersive interaction envelope and a more compact network of electrostatic and hydrogen bonds. For the most effective selector, SF-β-CD, the complexation Gibbs free energy (ΔG) in an aqueous medium is −29.5 kcal/mol for (+)-[S]-BUN versus −17.1 kcal/mol for (−)-[R]-BUN. This results in a large free energy difference (ΔΔG) of 12.4 kcal/mol, indicating exceptional enantioselectivity. The NCI isosurfaces also confirm that the S-isomer achieves optimal stereoelectronic complementarity with the host’s sulfate and hydroxyl groups, showing lower steric penalties than the R-isomer. These findings provide a robust prediction of a longer electrophoretic migration time for the (+)-[S]-enantiomer and validate SF-β-CD as a highly efficient chiral selector for BUN, underscoring the power of in silico methods to elucidate complex chiral recognition mechanisms.

**Methods:**

Semiempirical geometry, frequency, non-covalent, 2nd version, eXtended Tight Binding (GFN2-xTB) and Density Functional Theory (DFT) (ωB97X-D3/6-31G(d,p) and ωB97X-D3/6–311 + G(d,p)) methods were employed in both gas and aqueous phases. NCI analysis was also performed. All DFT and semiempirical calculations were carried out using the ORCA 5.0 software package. The NCI analysis was carried out using the Multiwfn program.

## Introduction

**Fig. 1:**
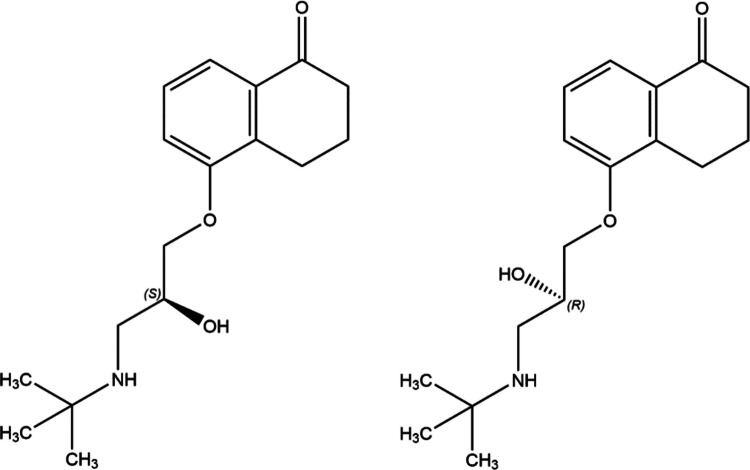
Chemical structures of levobunolol enantiomers

Levobunolol (BUN, Fig. 1), a non-selective β-adrenergic antagonist, is a topical ophthalmic agent used for the management of intraocular pressure in patients with open-angle glaucoma and ocular hypertension [1, 2]. The pharmacological activity of BUN is primarily attributed to the (+)-(S)-enantiomer, which possesses significantly greater β-blocking potency than its (−)-(R)-antipode [3]. The differential bioactivity between enantiomers—a common feature of chiral drugs—underscores the importance of enantioselective separation. Such resolution is essential to ensure the administration of the therapeutically active isomer, thereby maximizing efficacy while minimizing potential adverse effects.

Several techniques can be used to resolve the enantiomers of chiral compounds, most notably liquid and gas chromatography, which rely on chiral stationary phases [4, 5]. However, capillary electrophoresis (CE) has emerged as a particularly powerful analytical method for chiral separations [6, 7]. It offers significant advantages, including high efficiency, rapid analysis times, low reagent and sample consumption, and suitability for miniaturization. A key feature of CE is its ability to use chiral selectors (CSs) in the mobile phase, which eliminates the need for a chiral stationary phase [8]. Additionally, CE is increasingly being used to investigate the structural basis of intermolecular interactions [9].

Enantiomeric resolution is achieved by dissolving the CSs in the background electrolyte (BGE). These selectors reversibly interact with enantiomers to form transient diastereomeric complexes of different stabilities, following a host–guest interaction model. This differential stability arises because the enantiomers’ distinct spatial structures result in varied binding affinities with the CS [10]. The complexation is governed by non-covalent forces like hydrogen bonding, van der Waals forces, and π-π stacking [11, 12]. Cyclodextrins (CDs) are premier examples of these selectors, known for their toroidal structure composed of a hydrophilic exterior and a hydrophobic internal cavity, which is ideal for forming such complexes [13, 14].

A deep understanding of the molecular interactions between enantiomers and CS is essential for predicting the enantiomer migration order (EMO) in multifactorial CE separations. Currently, the field faces a knowledge gap, as there is often a lack of detailed 3D structural data for the resulting diastereomeric complexes and a clear link between this structure and the EMO, hindering further progress [15]. Conventional methods for EMO determination rely on analytical techniques like circular dichroism, polarimetry, NMR spectroscopy, and X-ray diffraction, all of which require enantiopure standards [16]. These established techniques are frequently impractical due to their significant expense and the limited availability of the necessary pure materials. To bypass these challenges, computational modeling of diastereomeric complexes has become a promising alternative, offering a reliable method for EMO prediction by rigorously evaluating the structural, topological, electronic, and energetic characteristics of the entities involved [17].

Our 2021 review summarized research from 2016 to 2021 that employed both computational and experimental approaches to investigate chiral recognition in CE with CDs [18]. Many research teams have concentrated on understanding these mechanisms through various theoretical methods. The most common of these are classical molecular dynamics (MD) simulations, molecular docking, hybrid quantum mechanics/molecular mechanics (QM/MM) calculations, semiempirical methods (SEM), and density functional theory (DFT).

**Fig. 2:**
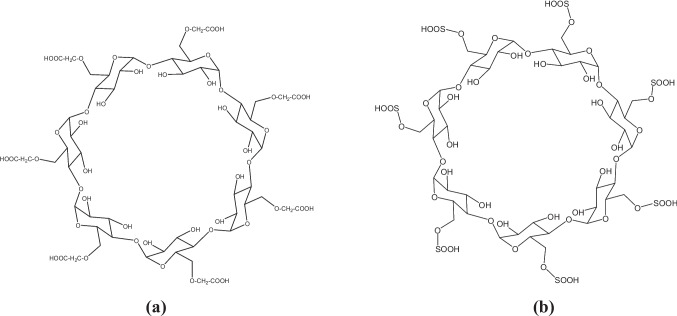
Chemical structures of carboxymethyl-β-CD (**a**) and sulfated-β-CD (**b**)

This study presents a computational investigation into the chiral recognition mechanism between BUN enantiomers and substituted β-cyclodextrins (β-CDs; Fig. 2).

By applying SEM and DFT calculations, we assessed the structural features and energetic properties of the resulting diastereomeric complexes to explain the observed EMO. The principal objective was to establish a quantitative, molecular-level understanding of this enantioseparation process. A comprehensive literature review confirmed a lack of theoretical research on this specific system, highlighting the novelty of our work.

## Theoretical methodology

Even with the increasing prevalence of DFT in recent decades, SEMs remain highly relevant due to their significant computational efficiency. It has been established in the literature that SEMs, such as PM3 and PM6, produce dependable estimations of molecular properties, especially molecular geometries. Therefore, their low computational cost makes them a suitable approach for characterizing diastereomeric complexes that arise during chiral capillary electrophoresis [18]. A more robust quantum mechanical framework is offered by DFT, which enables precise energetic calculations. However, for investigating host–guest interactions in such complex systems, a more balanced and powerful strategy is to integrate two methodologies. This hybrid approach first employs the computational speed of SEMs for geometry optimization, followed by energetic refinement using the higher accuracy of DFT. Our research group has previously applied this sequential semiempirical/DFT methodology with success to the study of supramolecular systems involving supramolecular complexes [19–24].

We initiated our investigation by performing unconstrained geometry optimizations for all relevant species using GFN2-xTB level of theory, employing the xTB code (version 6.4.1) [25]. These included the (−)-[R]-BUN and (+)-[S]-BUN enantiomers (Fig. 1), the carboxymethyl-β-CD (CM-β-CD) and sulfated-β-CD (SF-β-CD) chiral selectors (Fig. 2), and the four resulting host–guest complexes: (−)-[R]-BUN-CM-β-CD, (−)-[R]-BUN-SF-β-CD, (+)-[S]-BUN-CM-β-CD, and (+)-[S]-BUN-SF-β-CD. Subsequently, harmonic frequency calculations confirmed that all stationary points (isolated molecules and complexes) were true minima on the potential energy surface (PES), as indicated by exclusively real vibrational frequencies. Levobunolol was modeled exclusively in its neutral state throughout all calculations; protonated states of the amine moiety were not evaluated within the theoretical protocol adopted in this study. Therefore, the trends discussed herein should be interpreted within the context of the neutral-state model adopted in this work.

The isolated structures of BUN were obtained from a molecular database, whereas the cyclodextrin structures were retrieved from the laboratory’s structural database, having been previously validated in prior studies conducted by our research group. Based on these initial geometries, the guest–host arrangements were systematically generated using the UD-APARM approach.

The assembly of the 1:1 inclusion complex was performed using the UD-APARM (User-Defined Association Parameters) program [26], which systematically constructs molecular arrangements based on geometric and topological criteria. This method ensured a rational positioning of the drug within the host cavity. UD-APARM software was designed to generate and standardize the spatial arrangement of supramolecular associations. In this sense, the geometry of the isolated BUN molecule was obtained from crystallographic databases, ensuring a reliable structural representation of the drug prior to the complexation stage. From the generated initial structures, various supramolecular arrangements were evaluated in UD-APARM: the complexes corresponding to the lowest energies obtained under the adopted protocol. Following this stage, all selected complexes underwent full, unconstrained optimization and were confirmed as true minima through vibrational frequency analysis.

Unlike traditional methods that rely on qualitative descriptions, this software uses the principal axes of inertia of each molecule to establish a rigorous geometric reference. While molecular dynamics traditionally relies on molecular mechanics (force fields) to explore the potential energy surface, the methodology involving UD-APARM allows for the systematic construction and subsequent optimization of complexes using SEM methods. For more details about UD-APARM see ref[26].

In order to obtain more accurate energetic values, the geometries derived from the SEM calculations were subjected to a further round of single-point energy calculations. We utilized the ωB97X-D3 functional [27] with the 6-31G(d,p) and 6–311 + G(d,p) basis sets within the DFT framework, considering environments in both the gas phase and aqueous solution.

The complexation Gibbs free energy (ΔG) between the chiral selectors and the BUN enantiomers, a key parameter for understanding the EMO, was calculated using Eq. 1. This equation stems from the framework of statistical thermodynamics [28] and combines the variation in electronic energy (ΔE) with the thermal contributions to the Gibbs free energy (ΔG_T_) for the complex formation reaction.

1 $$\Delta G= {\Delta E+\Delta G}_{T}$$

It is important to clarify that the complexation Gibbs free energy (ΔG) values reported in this study should not be interpreted as absolute binding free energies directly comparable to experimental thermodynamic data. Instead, these values represent relative stabilization free energies obtained within a consistent computational protocol, combining DFT single-point electronic energies with harmonic thermal corrections and implicit solvation effects [29].

Accordingly, the ΔG values are employed primarily to compare relative stability trends between host–guest complexes, rather than to reproduce quantitative experimental free energies.

To account for the effects of water as a solvent, we employed the solvation model based on density (SMD) [30]. In this implicit solvation framework, the aqueous medium is treated as a continuum defined by the dielectric constant of water (*ε* = 78.4). The model situates the solute molecule within a custom-fit cavity, which is then immersed in this dielectric continuum. It should be emphasized that this choice corresponds to a methodological decision, and that the reported energetic values reflect relative stabilization trends in solution, rather than structures explicitly optimized in the solvent.

The Multiwfn 3.8 software [31] was used to calculate the non-covalent Interactions (NCI) analysis, which were visualized using VMD 1.9 [32]. All DFT and SEM calculations were carried out using the ORCA 5.0 software package [33].

## Results and discussions

### Thermodynamic analysis

To elucidate the molecular foundations governing the EMO, we performed a systematic investigation of the thermodynamic properties of the inclusion complexes. By employing high-level computational modeling to determine binding affinities, we established a robust rationale for the observed differences in electrophoretic mobility. The resulting data provide a molecular-level interpretation of the relative electrophoretic behavior of the enantiomers, supporting a proposed assignment of each enantiomer to its corresponding peak in the electropherogram. In the following analysis, the calculated ΔG values are discussed in a comparative manner, focusing on the relative stabilization between the enantiomeric complexes. Considering the absence of experimental studies available in the literature for this system, the present results may serve as a theoretical reference and guidance for future experimental investigations. In the following analysis, the calculated ΔG values are discussed in a comparative manner, focusing on the relative stabilization between enantiomeric complexes. Therefore, the absolute magnitudes should be interpreted within the context of the adopted level of theory. Within this context, the relative ΔG differences are used to support the discussion of chiral recognition and the proposed enantiomer migration order.

Table 1 presents the aqueous phase ΔG values obtained at the ωB97X-D3/6-31G(d,p)//GFN2-xTB and ωB97X-D3/6–311 + G(d,p)//GFN2-xTB levels of theory for the complexes formed between the BUN enantiomers, (−)-[R]-BUN and (+)-[S]-BUN, and the chiral selectors, CM-β-CD and SF-β-CD. These complexes are designated as (−)-[R]-BUN-CM-β-CD, (−)-[R]-BUN-SF-β-CD, (+)-[S]-BUN-CM-β-CD, and (+)-[S]-BUN-SF-β-CD.

**Table 1:** Complexation Gibbs free energy (ΔG), obtained at the ωB97X-D3/6-31G(d,p)//GFN2-xTB and ωB97X-D3/6–311 + G(d,p)//GFN2-xTB levels of theory, for the inclusion complexes formed between BUN enantiomers and the chiral selectors, considering water as the solvent. Values are reported in kcal mol^−1^

Complexes	ωB97X/6-31G(d,p)//GFN2-xTB	ωB97X/6–311 + G(d,p)//GFN2-xTB
	ΔG	ΔG
(−)-[R]-BUN-CM-β-CD	−15.2	−17.4
(+)-[S]-BUN-CM-β-CD	−22.7	−26.8
(−)-[R]-BUN-SF-β-CD	−17.1	−20.9
(+)-[S]-BUN-SF-β-CD	−29.5	−33.2

The data in Table 1 indicates that complexes involving the (+)-[S]-BUN enantiomer were consistently more stable. Notably, for CM-β-CD, the theoretical prediction that the S-enantiomer migrates after the R-enantiomer is in good agreement with previously reported experimental data [34]. However, to the best of our knowledge, no experimental data are currently available for the SF-β-CD system. In the next sections, a detailed investigation of the molecular-level structures will provide an explanation for this stability difference.

As can be seen in Table 1, the single-point energy calculations performed with the more robust 6–311 + G(d,p) basis set—which includes diffuse functions—yielded more negative ΔG values across all evaluated systems. However, the energetic trends remain remarkably systematic and fully consistent with the results obtained using the smaller 6-31G(d,p) basis set. Most importantly, the stereoselective preference is perfectly preserved regardless of the basis set employed: the (+)-[S]-BUN enantiomer consistently exhibits a significantly stronger binding affinity (more negative ΔG) than the (−)-[R]-BUN enantiomer for both CM-β-CD and SF-β-CD hosts. These findings confirm that while a larger basis set refines the absolute interaction energies, the double-zeta 6-31G(d,p) basis set is already reliable for accurately describing the relative trends and the qualitative nature of the host–guest interactions.

Additionally, from the Gibbs free energy values presented in Table 1, the difference in ΔG (ΔΔG) was calculated according to Eq. 2, with the results provided in Table 2.

2 $$\Delta \Delta G=\left|{\Delta G}_{\left[R\right]}\right|-\left|{\Delta G}_{\left[S\right]}\right|$$

**Table 2:** Gibbs free energy difference (ΔΔG) values obtained at ωB97X-D3/6-31G(d,p)//GFN2-xTB level of theory, for the chiral selectors CM-β-CD and SF-β-CD. Values are reported in kcal mol^−1^

Chiral selector	ΔΔG
	ωB97X-6-31G(d,p)//GFN2-xTB
CM-β-CD	7.5
SF-β-CD	12.4

It is important to mention that the calculated ΔΔG values are based on thermodynamic criteria. In the literature, free energy differences of ca. 2.0 kcal mol^−1^ are considered sufficient to enable enantiomeric separation [35]. Herein, depending on the chiral selector, the values ranged from 7.5 to 12.4 kcal mol^−1^ (Table 2), indicating that both selectors show potential for enantiomeric discrimination. The chiral selector SF-β-CD, in particular, exhibited the highest ΔΔG values, suggesting greater efficiency in the enantioseparation of BUN.

### Structural analysis

#### CM-β-CD complexes

**Fig. 3:**
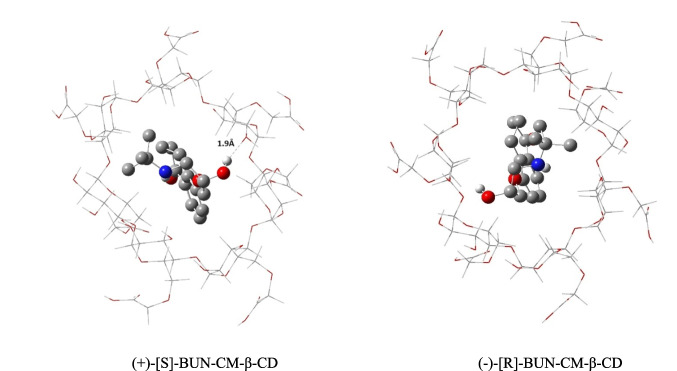
Inclusion complexes of BUN enantiomers with CM-β-CD. Hydrogen bonds are depicted by dashed lines

As shown in Fig. 3, the complex formed with the (+)-(S) enantiomer adopts a more favorable orientation to interact with the available binding sites of the chiral selector. This spatial arrangement allows a more efficient accommodation of the guest within the cavity, promoting cooperative stabilization and enabling the formation of a short hydrogen bond (1.9 Å) between the selector and the enantiomer. In contrast, for the complex formed with the (−)-(R) enantiomer, as also depicted in Fig. 3, the optimized geometry does not exhibit a well-defined short hydrogen bond according to classical geometric criteria. Instead, the interaction pattern is characterized by a less cooperative arrangement of contacts within the cavity. This difference is reflected in a higher complexation energy and is further elucidated by the NCI analysis, which reveals the presence of localized repulsive regions and a less continuous attractive interaction network for the (R)-enantiomer.

Although this initial analysis provides a clear qualitative trend, it remains somewhat simplistic. To fully comprehend the energetic landscape of these supramolecular systems, a more robust quantitative assessment of all intermolecular interactions is required. The mere identification of a single hydrogen bond or the observation of steric proximity does not account for the delicate balance of non-covalent forces—such as dispersion, dipole–dipole, and π-π interactions—that collectively dictate chiral recognition.

To address this limitation and gain a deeper insight into the nature of these contacts, we performed an NCI analysis. This topological method allows for the visualization and quantification of interaction regions based on the electron density and its derivatives, specifically the reduced density gradient. By mapping these interactions, we can distinguish between attractive and repulsive forces, providing a comprehensive “fingerprint” of the stabilization mechanisms within the supramolecular cavity.

**Fig. 4:**
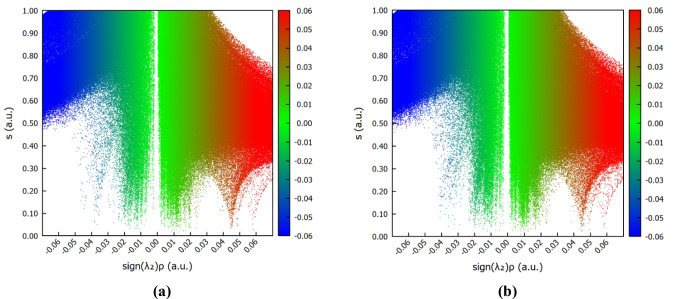
2D NCI plot for the complexes: BUN-(−)-R-CM-β-CD (**a**) and (+)-[S]-BUN-CM-β-CD (**b**)

The NCI analysis of the inclusion complexes formed between BUN enantiomers and CM-β-CD provides a high-resolution topographical map of the stabilizing forces involved. The 2D NCI plots for both the R-BUN and S-BUN complexes (Fig. 4) exhibit a characteristic dense concentration of points in the (λ₂)*ρ* ≈ 0 region across a broad range of the reduced density gradient (*s*), which is diagnostic of pervasive van der Waals interactions. These dispersive forces, arising from the steric fit of the BUN aromatic and aliphatic moieties within the relatively hydrophobic cavity of the CM-β-CD, act as the primary drivers for host–guest stabilization. Furthermore, both complexes display significant point populations at (λ₂)*ρ* < 0, corresponding to strong attractive interactions such as hydrogen bonding and electrostatic attractions between the BUN hydroxyl/amine groups and the carboxylate/hydroxyl substituents of the CM-β-CD.

**Fig. 5:**
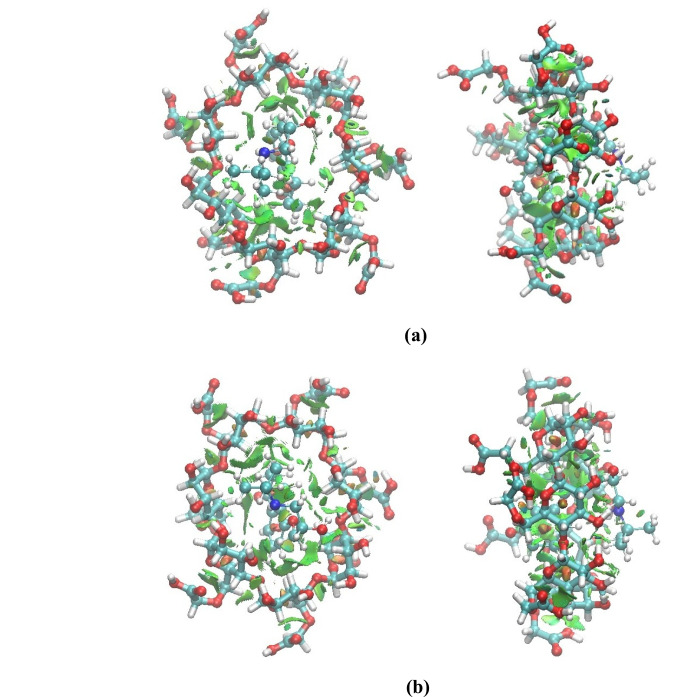
3D NCI isosurfaces for the complexes: BUN-(−)-R-CM-β-CD (**a**) and (+)-[S]-BUN-CM-β-CD (**b**)

The 3D NCI isosurfaces (Fig. 5) elucidate the spatial distribution of these interactions, revealing a distinct structural superiority in the (+)-[S]-BUN-CM-β-CD complex. While the R-enantiomer exhibits a fragmented “shell” of green isosurfaces, suggesting discontinuous hydrophobic contact, the S-enantiomer displays a significantly more continuous and uniform envelope. This indicates that the S-enantiomer achieves a more optimal spatial orientation, maximizing the contact area with the host’s inner cavity. Additionally, the attractive domains (blue regions) in the S-complex appear more compact and cooperative, reflecting a more robust network of hydrogen bonds. Conversely, the steric repulsion zones ((λ₂)*ρ* > 0, red regions) are less extensive for the S-enantiomer compared to the R-enantiomer, where bulky groups experience increased steric repulsion, as evidenced by localized repulsive regions in the NCI analysis.

The qualitative differentiation observed in the NCI profiles is corroborated by both the computed thermodynamic parameters in aqueous solution and the existing experimental benchmarks for BUN enantioseparation [33]. This synergy between topological analysis and energetic data reinforces the molecular rationale for the observed chiral discrimination. The (+)-[S]-BUN-CM-β-CD complex demonstrates a more pronounced stabilizing energy profile (ΔG = −22.7 kcal mol^−1^) relative to the R-BUN counterpart (ΔG = −15.2 kcal mol^−1^). The integrated analysis confirms that although the nature of the binding forces is identical for both enantiomers, the S-enantiomer’s superior enantioselective recognition is a direct result of a more favorable balance between maximizing dispersive and electrostatic attractions while simultaneously minimizing global steric penalties.

#### SF-β-CD complexes

In the system involving SF-β-CD the same behavior is observed. The S-enantiomer forms the more stable complex, exhibiting a complexation energy that is ca. 12.0 kcal/mol more negative than that of the R-enantiomer a difference that reflects a higher intermolecular affinity, as shown in Table 2. This enhanced stability can be attributed to the presence of sulfate groups at the cyclodextrin rim, which introduce anionic centers capable of establishing strong electrostatic interactions with polar moieties of the guest, as well as promoting directional hydrogen bonds.

**Fig. 6:**
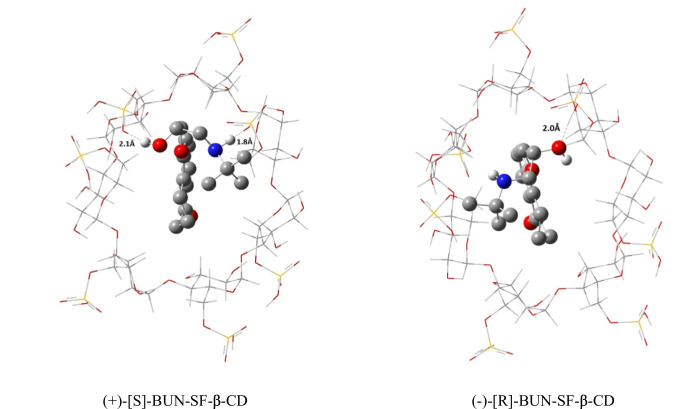
Inclusion complexes of BUN enantiomers with SF-β-CD. Hydrogen bonds are depicted by dashed lines

The spatial orientation of the S-enantiomer within the cavity of SF-β-CD allows for a deeper and more snug encapsulation, promoting the formation of a greater number of stronger hydrogen bonds (two–1.8 Å and 2.1 Å) (Fig. 6) with the sulfate and hydroxyl groups of the cyclodextrin in comparison to the R-enantiomer. Whereas the S-enantiomer positions itself to maximize contact points with the host, cooperatively exploiting the available anionic regions, the R-enantiomer exhibits a less compatible geometry, resulting in more dispersed interactions (Fig. 6). This enhanced stereoelectronic complementarity of the S-enantiomer reduces steric repulsions and lowers the energetic penalty for the conformational adjustments of both the drug and the cyclodextrin, thereby favoring a more stable, organized, and energetically favorable structure. These combined factors elucidate why the complex with the S-enantiomer presents a more exergonic complexation energy, indicative of superior thermodynamic stability that is directly related to the number of established hydrogen bonds.

**Fig. 7:**
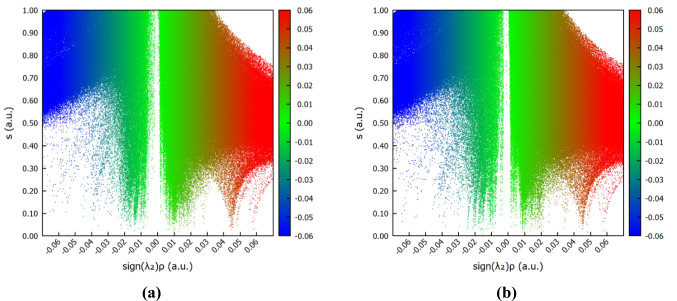
2D NCI plot for the complexes: BUN-(−)-R-SF-β-CD (**a**) and (+)-[S]-BUN-SF-β-CD (**b**)

The 2D NCI analysis for both R-BUN and S-BUN complexes (Fig. 7) with SF-β-CD reveals a stabilization profile governed by a competitive equilibrium between dispersive, electrostatic, and steric repulsive forces. The plots exhibit a significant concentration of points near (λ₂)*ρ* ≈ 0, characteristic of extensive van der Waals contacts between the hydrophobic interior of the cyclodextrin cavity and the non-polar moieties of the guest. Notably, a dense population in the (λ₂)*ρ* < 0 region indicates strong attractive interactions, primarily driven by electrostatic contributions between the sulfonate groups of the host and the amine group of BUN, further supplemented by hydrogen bonding involving hydroxyl groups. Conversely, the presence of signals at (λ₂)*ρ* > 0 denotes localized steric repulsion and repulsive regions associated with the bulky sulfonate substituents, indicating that stabilization occurs despite the presence of unfavorable spatial interactions within the highly functionalized cavity.

**Fig. 8:**
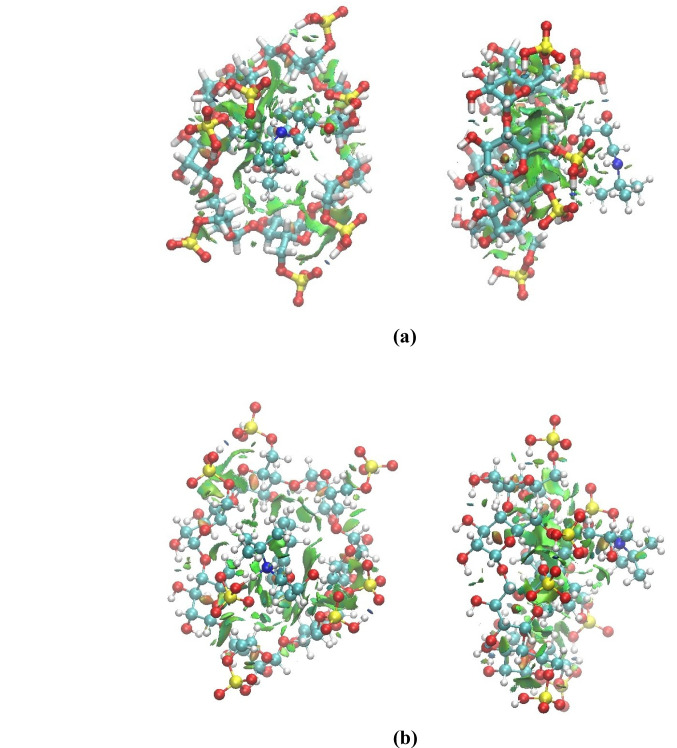
3D NCI isosurfaces for the complexes: BUN-(−)-R–SF-β-CD (**a**) and (+)-[S]-BUN-SF-β-CD (**b**)

3D NCI isosurfaces (Fig. 8) provide spatial clarity on these interactions, demonstrating that while both enantiomers occupy the host cavity with their aromatic rings oriented toward the hydrophobic core, the supramolecular organization differs significantly between them. For the BUN-(+)-R complex, the green isosurfaces representing dispersive interactions appear segmented and discontinuous, suggesting that the hydrophobic covering provided by the SF-β-CD cavity is not fully optimized. Although strong attractive domains are present near the polar groups, they appear less compact and spatially disorganized, indicating a lack of ideal geometric complementarity. This spatial arrangement is further constrained by compressed NCI surfaces, which confirm localized steric repulsion between the guest and the bulky substituents of the host.

In contrast, the (+)-[S]-BUN-SF-β-CD complex exhibits a superior stereoselective fit, characterized by a more continuous and homogeneous envelope of dispersive isosurfaces surrounding the guest. This suggests that the S-enantiomer maximizes the available hydrophobic surface area more efficiently than its R-counterpart. Furthermore, the attractive domains associated with electrostatic interactions and hydrogen bonding are more compact and well-defined, pointing toward a more cooperative and organized interaction network with the sulfonate and hydroxyl groups. The reduction in the extent of compressed NCI surfaces for the S-enantiomer indicates a lower overall steric penalty, leading to a more thermodynamically favorable and geometrically optimized inclusion complex compared to the R-enantiomer.

## Conclusions

In this study, we employed ωB97X-D3/6-31G(d,p)//GFN2-xTB and ωB97X-D3/6–311+G(d,p)//GFN2-xTB calculations to investigate the enantioselective separation of BUN enantiomers through the formation of inclusion complexes with substituted β-CD.

Our analysis of the complexes’ energetic and structural properties provided molecular-level insights into the EMO. Overall, the S-enantiomer formed more stable complexes, which suggests a more prolonged migration time compared to R-enantiomer It should be noted that the energetic differences discussed in this work correspond to relative stabilization trends obtained within the adopted theoretical methodology and are not intended to reproduce experimental binding free energies.

The NCI index analysis, applied in both 2D and 3D frameworks, reveals that the stabilization of inclusion complexes between BUN enantiomers and the modified cyclodextrins CM-β-CD and SF-β-CD is governed by a synergistic interplay of predominant dispersive contacts, electrostatic/hydrogen-bonding interactions, and localized steric repulsive contributions. In this context, steric effects are discussed as topological features revealed by the NCI analysis, rather than as results of a quantitative energetic evaluation. For both hosts, the S-enantiomer demonstrates a more effective exploitation of the cyclodextrin cavity compared to its R-counterpart. This superior molecular fit supports a proposed enantiomer migration order for CM-β-CD, indicating that the S-enantiomer is expected to display stronger interactions with the chiral selector and, consequently, a longer electrophoretic migration time. This is evidenced by 3D NCI maps showing a more homogeneous dispersive envelope and more compact attractive domains, which correlate directly with the more negative Gibbs free energy (ΔG) calculated for the S-enantiomer.

Furthermore, the high ΔΔG values point to an excellent potential for enantiomeric separation, for which SF-β-CD is remarkably effective. Consequently, our findings indicate that SF-β-CD is a viable selector for the enantioselective analysis of BUN.

It should be emphasized that the proposed enantiomer migration order is based on thermodynamic considerations derived from relative complex stabilization. Other factors that influence electrophoretic migration, such as selector concentration, charge state, complex mobility, and kinetic effects, are not explicitly addressed in this study. In the absence of experimental EMO data for both CM-β-CD and SF-β-CD systems, the present analysis should be interpreted as a qualitative and interpretative framework rather than a quantitative description of electrophoretic behavior.

In summary, this study highlights the robust predictive power of computational chemistry in elucidating enantioselective processes. By characterizing the inclusion complexes at the molecular level, we successfully predicted the migration order and identified the specific enantiomer with greater retention within the electrophoretic system.

## Data Availability

No datasets were generated or analysed during the current study.
